# Exploring the Potential of Invasive Species *Sargassum muticum*: Microwave-Assisted Extraction Optimization and Bioactivity Profiling

**DOI:** 10.3390/md22080352

**Published:** 2024-07-30

**Authors:** Aurora Silva, Lucia Cassani, Maria Carpena, Catarina Lourenço-Lopes, Clara Grosso, Franklin Chamorro, Pascual García-Pérez, Ana Carvalho, Valentina F. Domingues, M. Fátima Barroso, Jesus Simal-Gandara, Miguel A. Prieto

**Affiliations:** 1 Instituto de Agroecoloxía e Alimentación (IAA)—CITEXVI, Nutrition and Bromatology Group, Department of Analytical Chemistry and Food Science, Universidade de Vigo, 36310 Vigo, Spain; mass@isep.ipp.pt (A.S.); luciavictoria.cassani@uvigo.es (L.C.); mcarpena@uvigo.es (M.C.); c.lopes@uvigo.es (C.L.-L.); franklin.noel.chamorro@uvigo.es (F.C.); pasgarcia@uvigo.es (P.G.-P.); jsimal@uvigo.es (J.S.-G.); 2 REQUIMTE/LAQV, Instituto Superior de Engenharia do Porto, Instituto Politécnico do Porto, Rua Dr António Bernardino de Almeida 431, 4249-072 Porto, Portugal; aclaragrosso@gmail.com (C.G.); vfd@isep.ipp.pt (V.F.D.); 3 Department for Sustainable Food Process, Università Cattolica del Sacro Cuore, Via Emilia Parmense 84, 29122 Piacenza, Italy; 4 Escola Superior de Biotecnologia, CBQF—Centro de Biotecnologia e Química Fina—Laboratório Associado, Universidade Católica Portuguesa, Rua Diogo Botelho 1327, 4169-005 Porto, Portugal; apcarvalho@ucp.pt

**Keywords:** microwave-assisted extraction, antioxidants, bioactive compounds, *Sargassum muticum*

## Abstract

*Sargassum muticum* (SM) poses a serious environmental issue since it is a fast-expanding invasive species occupying key areas of the European shoreline, disrupting the autochthonous algae species, and disturbing the ecosystem. This problem has concerned the general population and the scientific community. Nevertheless, as macroalgae are recognized as a source of bioactive molecules, the abundance of SM presents an opportunity as a raw material. In this work, response surface methodology (RSM) was applied as a tool for the optimization of the extraction of bioactive compounds from SM by microwave-assisted extraction (MAE). Five different parameters were used as target functions: yield, total phenolic content (TPC); and the antioxidant measurements of 2,2-diphenyl-1-picrylhydrazyl radical scavenging activity (DPPH), 2,2′-azino-bis (3-ethylbenzothiazoline-6-sulfonic acid) diammonium salt (ABTS), and β-carotene bleaching (BC). After the optimal extraction conditions were determined (time = 14.00 min; pressure = 11.03 bar; ethanol = 33.31%), the chemical composition and bioactivity of the optimum extract was evaluated to appraise its antioxidant capability to scavenge reactive species and as a potential antibacterial, antidiabetic, antiproliferation, and neuroprotective agent. The results lead to the conclusion that MAE crude extract has bioactive properties, being especially active as an antiproliferation agent and as a nitric oxide and superoxide radical scavenger.

## 1. Introduction

The number of marine macroalgae introduced into non-native ecosystems has drastically increased due to market globalization, global warming, and other economic activities such as aquaculture and tourism. These invasive species are defined as exotic or non-native species when they have been introduced in habitats different to their own or with unusual abundance and can cause a serious environmental impact by reducing the autochthonous biodiversity and causing a shift in trophic networks in the marine ecosystems that they colonize [1,2]. Alongside this ecological impact, the increased presence of invasive species also influences the economy as it affects the fishing industry, more precisely, fishermen’s equipment such as boats and nets.

Despite being an invasive brown macroalga from Japan, *Sargassum muticum* (Yendo) Fensholt (SM) is among the most prevalent *Sargassum* species on European coastlines [3]. This high pervasiveness can be explained by its monoecious characteristics allowing it to disperse reproductive fronds that spread up to 5 m to find another fertile alga, making it an extremely fast and efficient opportunist species [4,5,6]. SM occupies preferably tidal pools, where the water remains at low tide, and can form remarkably dense populations from spring to late summer in the coastal fringe, with depths of 10 m [4]. Several attempts to eradicate SM have been developed, but with little to no success. Consequently, seasonal harvesting of the alga was adopted [5]. This raw material was once often collected since it was utilized as fertilizer. However, because it requires manual labor and is reliant on the tide, this method was abandoned, not being able to compete with more modern, less expensive fertilizers [6].

So, there is a massive availability of SM material that is not currently being utilized but may have great potential since brown algae species are known to have compounds with biological properties [7]. Furthermore, with the growing interest in environmentally friendly materials there is an increased search for natural supplies, and brown macroalgae have been identified as a rich source of bioactive compounds with antitumor, anti-inflammatory, antioxidant, antidiabetic, antiproliferation, and antimicrobial activities [7,8,9,10,11,12]. In this sense, brown macroalga SM can fill the gap in natural bioactive compounds and be employed as an ingredient or functional food [8,13,14]. So, while it is critical to manage SM expansion sustainably, this species can provide a high-value raw material, contributing to the management of the ecosystem.

As a result, macroalgae, notably the invasive SM, have gained the interest of the scientific community and several high-profile companies. Several green alternative techniques have been used to extract bioactive compounds from brown macroalgae, namely, the microwave-assisted extraction (MAE) technique [15,16]. MAE presents several advantages over traditional extraction techniques, such as a short extraction time, low solvent consumption, selective heating, high extraction efficacy, and limited degradation of the desired compounds [14]. Response surface methodology (RSM) was chosen to optimize the MAE process. It is a well-established method for optimizing various processes, including extraction, drying, blanching, enzymatic hydrolysis, clarification, etc. [17].

Under this framework, and focusing on adding value to this alga, this study aimed to explore the antimicrobial, antioxidant, neuroprotective, antidiabetic, and antiproliferation effects of SM. RSM was used as a strategy to optimize MAE extraction yield, the total phenolic content (TPC), and the antioxidant capacity by DPPH and ABTS^•+^- scavenging activity, and β-carotene bleaching assay. Later, the optimized extract was characterized in terms of phenolic composition by HPLC MS/MS and used to test the mentioned biological properties [17].

As the antioxidant potential is associated with the control of excess reactive oxygen species (ROS), which are risk factors for numerous chronic and degenerative diseases, including cancer, respiratory, neurological, and digestive disorders [18], a series of bioactive tests were made in the optimized SM extract to explore its radical scavenging, and antimicrobial, neuroprotective, and antiproliferation activity.

## 2. Materials and Methods

### 2.1. Chemicals and Solutions

Carlo Erba Reagents S.A was used to acquire all solvents. Salicylic acid was obtained from Fisher Scientific in Leicestershire, UK, and hydrogen peroxide was purchased from Laborspirit in Lisbon, Portugal. The rest were purchased from Sigma-Aldrich (St. Louis, MO, USA, and Steinheim, Germany).

### 2.2. Algae Sampling and Preparation

In the winter season of 2019, Algamar (www.algamar.com accessed on 30 May 2024) manually collected samples of SM from the northwest coast of Spain, specifically Galicia. The collected samples (17 specimens) underwent a series of processing steps: sorting, classification, washing with tap water, and finally, lyophilization using a LyoAlfa10/15 system from Telstar, Thermo Fisher Scientific. After this, the samples were transformed into a fine powder using a blender and were stored at −80 °C until being used for extraction.

### 2.3. Optimization Procedure

#### 2.3.1. Microwave-Assisted Extraction

The microwave-assisted extraction (MAE) was performed using a multiwave-3000 (Anton-Paar, Germany) in closed vessels. Briefly, the lyophilized SM was extracted with a solvent mixture (ethanol: water), with a solid to liquid ratio of 30 g/L. After the extraction, the samples were immediately put in an ice bath for 5 min. Later, the extracted samples were centrifuged at 9000 rpm for 15 min. The liquid phase was filtered (0.22 µm) and used to determine the extraction yield, the TPC, and the antioxidant capacity [19].

#### 2.3.2. Experimental Design and Mathematical Modelling

A circumscribed central composite design (CCCD) with 5 levels was employed to optimize 3 independent variables: *time* (*t* or *X*_1_, 3 to 25 min), *pressure* (*P* or *X*_2_, *2* to *20* bar), and *ethanol concentration* (*S* or *X*_3_, 0 to 100%). This design generated 28 combinations of responses: twenty-two experiments were established by the interplay of these variables and six replicates of the central point (Table 1). There were five response variables, all quantified in dry weight (dw): *Y*_1_ (mg/g dw), that represents the extraction yield; *Y*_2_ (mg PGE/g dw), the TPC; *Y*_3_ (nM R•/g dw), the DPPH; *Y*_4_ (nM R•/g dw), the ABTS^•+^-RSA; and *Y*_5_ (µM BC/g dw), the BCM. The data were fitted to a polynomial model using the least squares regression technique, as indicated in Equation (1):

(1) $$Y=b_{0}+\sum_{i=1}^{n}b_{i}X_{i}+\sum_{\begin{array}{c} i=1\\ j>i \end{array}}^{n-1}\sum_{j=2}^{n}b_{ij}X_{i}X_{j}+\sum_{i=1}^{n}b_{ii}X_{i}^{2}+\sum_{\begin{array}{c} i=1\\ j>i \end{array}}^{n-1}\sum_{j=2}^{n}b_{iijj}X_{i}^{2}X_{j}^{2}+\sum_{\begin{array}{c} i=1\\ j>i \end{array}}^{n-2}\sum_{j=2}^{n-1}\sum_{\begin{array}{c} k=3\\ k>j \end{array}}^{n}b_{ijz}X_{i}^{2}X_{j}^{2}X_{k}^{2}+\sum_{i=1}^{n}b_{iii}X_{i}^{3}$$

where *Y* represents the dependent variable (response variables *Y*_1_ to *Y*_5_) and *X_i_* and *X_j_* are independent variables. On the other hand, *b*_0_ and *b_i_* correspond to the constant and linear effect coefficients, respectively; *b_ij_*, *b_ii_*, *b_iijj_*, and *b_iii_* are the linear interactive, quadratic, quadratic interactive, and cubic effects between the response variables, respectively; and *n* is the number of variables.

#### 2.3.3. Response Variables

##### Extraction Yield

The extraction yield was calculated as the relation between the dry weight of the crude extract and the mass of lyophilized alga used in each extraction point in mg/g. Briefly, crucibles were prepared (104 °C, 1–2 h) and weighed. Then, 5 mL of the extracted solution was added and put in the oven for 24 h (TCF forced air oven, Argo lab). After that time, the crucibles were cooled down in the desiccator and weighed to obtain the extraction yield.

##### Phytochemical Content

The total polyphenol content (TPC) of SM extracts was assessed using the Folin–Ciocalteu method with no modifications [20]. The results are given in mg of phloroglucinol equivalent (PGE)/g dw. All the tests were conducted in triplicate.

##### Antioxidant Activity

DPPH: The antioxidant capacity based on this method was performed based on a previously published work [21]. The results are expressed in nM DPPH/g extract dw.

ABTS^•+^ assay: This technique was carried out as described by Viacava et al. [22]. The results are expressed in nM ABTS^•+^/g alga extract dw.

β-Carotene bleaching method (BCM): This methodology was described previously [23]. The results’ units are in µM BC/g alga extract dw.

All the microscale analytical determinations were made in a Multi-Modal Synergy ™ HTX microplate reader at least in triplicate.

#### 2.3.4. Statistical Evaluation

All the statistical results, fitting procedures, and coefficient values were calculated using a Microsoft Excel spreadsheet. DeltaGraph v7 was used to create the graphic illustrations from the obtained data. The statistical evaluation of the experimental results for the optimization was performed as follows: (a) *Coefficient determination*: minimizing the sum of the quadratic differences between the obtained and predicted values to obtain the parametric estimates, using the least squares method (quasi-Newton) by the “solver” macro in Microsoft Excel; (b) *Coefficients’ significance*: the confidence intervals were calculated using “SolverAid” to obtain the coefficients’ significance, discarding the non-statistically significant terms for the *p*-value (*p* > 0.05); (c) *Model consistency*: Fisher’s F test (α = 0.05) was used to determine if the built models correctly described the obtained data; and (d) *Other statistical evaluation criteria*: “SolverStat” (prediction uncertainties of parameters and models) and the *R*^2^ value were used to confirm the homogeneity of the model (percentage of adaptability of each dependent variable explained by the model).

### 2.4. Chemical Characterization by HPLC-ESI-QqQ-MS/MS

The analysis of the phenolic profile was carried out using high-performance liquid chromatography (Dionex Ultimate 3000UPLC+ system, Thermo Scientific, Waltham, MA, USA) coupled with a triple quadrupole tandem mass spectrometer (Thermo Scientific TSQ Quantis, San Jose, CA, USA) equipped with a working electrospray ion (ESI) source in negative/positive mode (HPLC-ESI-QqQ-MS/MS). Compounds were separated using a Phenomenex nucleosil C18 column (5 μm, 100 Å, 4.6 mm × 150 mm) thermostatic at 40 °C.

The solvents used were (A) 0.1% formic acid in water and (B) acetonitrile in the following elution gradients: 15% B (5 min), 15–35% B (5 min), 35–70% B (10 min), 70–90% B (4 min), 90–15% B (1 min), and 15% B (5 min). A flow rate of 0.3 mL/min was used and the sample injection volume was 20 μL. For mass detection, the following parameters were used as universal conditions: sheath gas—30 Ar; auxiliary gas—10 Ar; ion transfer tube temperature: 325 °C; and vaporizer temperature: 350 °C.

Mass analysis was performed using a selected reaction monitoring (SRM) system and parameters (precursor/product ion, retention time, collision energy, and RF lens voltage) were optimized for each compound. The chemical compounds detected were classified into the following classes: alkaloids, flavonoids, phenolic acids, flavonoids, tannins, and terpenes. The semi-quantification of these classes was determined according to the calibration curve of the following standards, respectively: caffeine, epigallocatechin, gallic acid, ferulic acid, phloroglucinol, and genipin. The results are expressed in mg per g of extract (mg/g E) and data acquisition was performed using the Xcalibur 4.1 software.

### 2.5. Evaluation of the Biological Properties of the Optimum Extract

#### 2.5.1. Antioxidant Activity (ROS and RNS)

##### Superoxide Antiradical Activity

To appraise the superoxide (O_2_^●–^) antiradical activity, the methodology described by Oliveira and colleagues [24] was adopted. SM extracts were dissolved in KH_2_PO_4_/K_2_HPO_4_ buffer (19 mM; pH 7.4). Half-maximal inhibitory concentration (IC_50_, µg/mL) values were determined to express the results.

##### Hydrogen Peroxide Scavenging Activity

The H_2_O_2_ scavenging ability was determined based on the decrease in the signal at 230 nm, according to previous methods [25,26]. A blank sample was made for each dilution by replacing H_2_O_2_ solution with the buffer, and buffer solution was used as the negative control. The results are expressed as IC_50_ (µg/mL).

##### Hydroxyl Antiradical Scavenging Activity

Hydroxyl radical scavenging (^●^HO) was performed based on the salicylic acid method [27], as described formerly, with some modifications [28]. Sample blanks were prepared by replacing H_2_O_2_ with demineralized water, and the alga extract with water in negative controls. The results are expressed as IC_50_ (µg g/mL).

##### Nitrogen Oxide Antiradical Scavenging Activity

^●^NO scavenging activity was estimated based on a diazotization reaction [29,30]. Briefly, six different concentrations of SM extract were tested. Buffer alone was used as a negative control, and 2% phosphoric acid was added to the blank. The results are expressed as IC_50_ (µg/mL). Ascorbic acid was used as the reference control for all antioxidant assays.

#### 2.5.2. Antimicrobial Activity Assay

##### Microorganisms and Cultures

Active cultures of the Gram-positive bacteria *Staphylococcus aureus* (ATCC 25923), *Staphylococcus epidermidis* (NCTC 11047), and *Bacillus cereus* (ATCC 14579), and Gram-negative bacteria *Pseudomonas aeruginosa* (ATCC 10145), *Salmonella enteritidis* (ATCC 13076), and *Escherichia coli* (NCTC 9001), were grown in Mueller–Hinton broth (MHB) overnight at 37 °C. After, the inoculum concentration was set to 0.1 ± 0.01 optical density at 600 nm (0.5 MacFarland standard) by dilution in fresh MHB [31].

##### Extract Preparation

The optimum extract of SM was lyophilized and dissolved in dimethyl sulfoxide (DMSO) to a 20 mg/mL concentration. Later, the solubilized extract was disinfected by filtration (0.20 µm sterile syringe filter).

##### Minimal Inhibitory Concentration

The minimal inhibitory concentration (MIC) test was performed by the microdilution method, adapted from previously described work [31]. Sample blanks, sterile medium with extract, positive controls prepared with inoculated medium, and negative controls with lactic acid (40%) were included in each test. An inhibition halo assay was performed following the methodology previously described with no adaptations [32].

#### 2.5.3. Enzyme Inhibition Assays

##### Cholinesterase Inhibition Assay

The acetylcholinesterase (AChE) and buthyrylcholinesterase (BuChE) inhibition methodology was firstly reported by Ellman et al. and based on the measurement of the thiocholine released during the acetylthiocholine/butyrylthiocoline hydrolysis under the influence of AChE or BuChE, respectively [33,34]. Galantamine was used as a positive control and buffer as a negative control. The results are expressed as IC_50_ µg/mL.

##### Monoamino Oxidase A and B Inhibition Assay

Monoamine oxidase A (MAO-A) and B (MAO-B) inhibition activity promoted by SM extracts was evaluated by measuring the production of 4-hydroxyquinoline at 314 nm for 70 min, using kynuramine (3.75 mM) as the substrate, according to previously published work [30,35]. Clorgyline was the positive control whereas negative controls and blanks were prepared by substituting extracts or enzymes with buffer, respectively. The results are expressed as IC_50_ µg/mL.

##### Tyrosinase Inhibition Assay

Tyrosinase inhibition was determined in accordance with Masuda et al. [36]. Blanks were prepared by substituting L-DOPA with buffer and kojic acid was employed as the test validation control. The results are expressed as IC_50_ µg/mL.

##### α-Amilase Inhibition Assay

The analysis of α-amylase activity was performed according to previous methods [37]. Briefly, 100 μL of extract and 100 μL of 1% starch solution were incubated for 10 min at RT. A volume of 100 μL porcine pancreatic α-amylase (0.5 mg/mL) was added and samples were incubated for an additional 10 min. Samples, starch, and enzyme solutions were prepared in 20 mM phosphate buffer also containing 6 mM of NaCL at pH 6.9. After, 200 μL of dinitrosalicylic acid color reagent was added to stop the reaction (100 °C for 5 min). Samples were cooled to RT, and 50 μL of each sample and 200 μL of water were added to the microplate and read at 540 nm. Blank values were subtracted from each well and the results were compared with the control [37] and the results are expressed as IC_50_ µg/mL.

#### 2.5.4. Antiproliferative Activity

The sulforhodamine B (SRB) method was used to evaluate the antiproliferative activity following a previously described procedure [38]. The activity was tested against human tumor cell lines A549 (lung adenocarcinoma), HepG2 (hepatocellular carcinoma), and AGS (gastric adenocarcinoma), and a non-tumor cell line obtained from African green monkey kidney (Vero), and ellipticine was used as a positive control [39].

All the described biological property tests were made in at least triplicate and the microscale methods were carried out in a Synergy^HT^ (BioTek Instruments) microplate reader; the half-maximal concentration (IC_50_ or GI_50_) was calculated using the GraphPad Prism8 software after fitting the experimental data to the Weibull equation (Equation (2)). The normality of the data was determined by the Shapiro–Wilk test (*p* > 0.05), and fitting was performed at the 95% confidence level, with a correlation coefficient (*R*^2^) greater than 0.9 for all tested parameters [40].

(2) $$Y\left(x\right)=k\left[1-EXP\;\left(-Ln2{\left(\frac{x}{\tau }\right)}^{a}\right)\right]$$

where *a* represents the dose–response curve slope, *τ* the half-maximal concentration (IC_50_ or GI_50_), and *k* the asymptote.

## 3. Results and Discussion

### 3.1. Theoretical Response Surface Models and Statistical Verification

The CCCD and the corresponding values obtained for each response criterion for the 28 treatments under the various experimental MAE conditions are shown in Table 1. From this table, it is seen that higher yields of extraction (*Y_DW_*) were obtained for lower ethanol percentages (between 0 and 20%); the highest result was obtained for experimental run number 3 using shorter times, medium energy, and a lesser percentage of ethanol concentration. On the contrary, the lowest results were obtained when *X*_3_:*S* = 100% (runs 14, 16, 18, 20, and 22). For the phytochemical content (*Y_TPC_*), the highest result was also obtained for experimental run number 3, and generally, the highest amounts were found when *X*_3_:*S* = 20% (runs 1, 3, 5, and 7). The lowest results were observed in the axial points. For the antioxidant responses (*Y_DPPH_*, *Y_ABTS_*, and *Y_BCM_*), the results generally agreed, showing the lowest results for axial points and the best activity in central points, corresponding to mild conditions of the variables. Even without model fitting, this initial approximation provides insight into the effect of the variables on each response.

Later, using the experimental data of the 28 different combinations of conditions (denoted as *X*_1_:*t*, *X*_2_:*P*, and *X*_3_:*S*) utilized in the MAE, fitting to the polynomial models using Equation (1) was performed. On the other hand, the estimated parametric values for each response—yield, *TPC*, *DPPH*, *ABTS*, and *BCM*—and numerical statistical criteria were obtained and are presented in Table 2. Those coefficients reflected as non-significant (ns) were not considered for the model development and are not displayed. The responses shown in Table 2 were correlated with the three independent variables using the polynomial equation by considering only the statistically significant coefficients (*p* < 0.05) to build simplified non-linear equations for each response presented below (Equations (3)–(7)):

(3) $$Y_{DW}=379.21+20.70P-62.28S+14.13t^{2}-31.48S^{2}-11.38S^{3}-4.07t^{2}P^{2}S^{2}$$

(4) $$Y_{TPC}=41.62+3.18P-17.12S+5.24t^{2}-3.30S^{2}+4.72S^{3}-1.42t^{2}P^{2}S^{2}$$

(5) $$Y_{DPPH}=58.92+1.77P-10.72t^{2}-12.31P^{2}-14.25S^{2}+2.43t^{2}P^{2}S^{2}$$

(6) $$Y_{ABTS}=53.86+13.66S-10.26t^{2}-6.61P^{2}-13.05S^{2}-5.59S^{3}$$

(7) $$Y_{BCM}=0.070-0.013P-0.037S-0.011t^{2}-0.014P^{2}+0.009S^{2}+0.004P^{3}+0.011S^{3}$$

Overall, the linear, quadratic, and interactive effects were statistically significant (*p* < 0.05), with all the variables having representation in each equation, proving the adequacy of the selection. The cubic term was mostly used for describing the effect of *X_3_*:*S*. Regarding the statistical analysis, the quadratic regression model resulted in determination of the R^2^ coefficient (Table 2). All tested responses showed values between 0.80 and 0.93, confirming the fitting between the experimental and the regression models, explaining more than 80% of the variability.

### 3.2. Impact of the Extraction Variables on the Target Responses and Optimal Conditions

**Figure 1:**
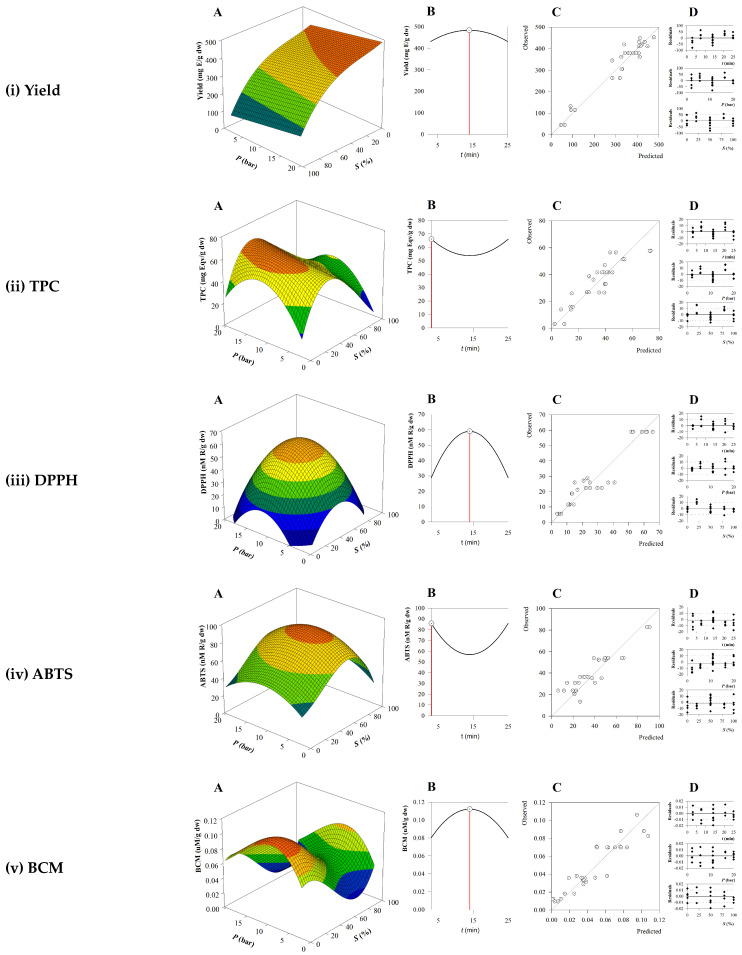
(**A**) Response surface plots of the combined effect of the two independent variables *X*_2_:*P* and *X*_3_:*S* while keeping *X*_1_:*t* at its central value. (**B**) Two-dimensional plot of the response variable as a function of time (*X*_1_:*t*). (**C**) Quadratic regression model of the predicted versus the experimental values. (**D**) Distribution of residual values.

The three-dimensional surface plots originating from the RSM analyses are represented in Figure 1A. In these plots, the interactive effect of the two independent variables (*X*_2_:*P* and *X*_3_:*S*) is shown, while the third (*X*_1_:*t*) remains at an intermediate value. Considering the interactive effect of the two independent variables, solvent concentration (*X*_3_:*S*) was the factor that most impacted the responses, indicating that lower ethanol percentages led to higher responses, suggesting that most of the extracted compounds and those with antioxidant activity were highly polar. Generally, medium to higher pressure values also resulted in increased responses. Figure 1B depicts two-dimensional plots as a function of time (*X*_1_:*t*), thus representing how the response is modelled through time and at which point the response is optimized for each response—yield, TPC, DPPH, ABTS, and BCM. From these results and in terms of response evolution through time, the optimum values were obtained for short to medium times. Figure 1C shows the regression model fitting the predicted and experimental values and in Figure 1D the distribution of residuals is shown. The randomized distribution of these points illustrates the absence of autocorrelation, and thus, the efficacy of the proposed models (Equations (3)–(7)). RSM was used to discover the MAE optimal extraction conditions (Table 2B) that maximize each of the studied responses—yield, TPC, DPPH, ABTS, and BCM—based on their specific model. Based on these results, the conditions that maximize yield aligned with the other responses in terms of time and pressure. However, maximum yield was obtained when using only water as the solvent, probably due to the high affinity and content of polysaccharides in the sample. Regarding the optimal conditions to maximize each antioxidant activity, all preferred medium pressure and short to medium times but the solvent was markedly different. This discrepancy originates from the different mechanisms of the antioxidant assays: single-electron transfer (SET) assays, namely, DPPH and ABTS, based on the donation of an electron, differ from hydrogen atom transfer (HAT) assays, namely, BCM, that donate a hydrogen atom [41]. Therefore, the ratio EtOH:H_2_O can affect the nature of the extracted molecules, and thus, the outcome of the response. For example, the BCM assay is adequate for measuring molecules with lipophilic properties, thus hydroethanolic mixtures are preferred [23]. TPC’s optimal conditions were aligned with antioxidant assays, confirming that this parameter is usually used to estimate the antioxidant capacity given their good correlation. Moreover, the differences between the responses were desired, to offer a complementary insight and contribute to overall comprehension of the governing mechanisms in SM compounds. At the optimal extraction point, the TPC value was 66.10 mg PGE/g dw (Table 2B). Several reports have described the measurement of TPC in SM crude extracts; nevertheless, a straightforward comparison between these reports is extremely hard to achieve since there are a multiplicity of factors influencing the TPC results, like seasonality [42], harvest location, and weather [3], in addition to extraction conditions. For instance, a study performed by Namvar and collaborators reported a TPC value in SM extract of 78.95 mg GAE/100 g dw [43]. Another report showed that TPC values for water and ethanol heat-assisted extracts (HAEs) of SM, were 230.8 and 114.9 mg GAE/g, respectively [44]. Furthermore, another study, that used a combination of enzymatic and ultrasound-assisted extraction (UAE) techniques, led to TPC values between 201 and 301 μg catechol equivalent/g lyophilized extract [45]. These differing findings support using optimization tools to find the extraction conditions that lead to higher yields and effectiveness of the extracts.

Within this framework, simultaneous optimization was developed leveraging RSM to find the highest possible recovery of phytochemical compounds with antioxidant properties from the SM extract. Our approach prioritized maximizing the outcomes projected by the established models. Consequently, the ideal conditions for MAE were identified as follows: time of 14.0 min (*t*), pressure of 11.03 bar (*P*), and ethanol concentration of 33.31% (*S*). Accordingly, the predicted values for each response under these optimal conditions were determined and are depicted below in Table 2B. These results suggest that achieving the best condition to maximize one particular response might negatively impact other responses. This underscores the importance of adopting simultaneous optimization methods.

### 3.3. Chemical Characterization by HPLC-ESI-QqQ-MS/MS

After determining extraction conditions that simultaneously maximize yield, TPC, and antioxidant capacity, a series of tests were performed to characterize the optimum extract from the chemical point of view. Therefore, based on the literature and knowledge about the biological properties of phenolic compounds present in brown algae, the sample was analyzed to identify and quantify these compounds [46,47,48,49,50,51,52].

This task is especially demanding in the case of macroalgae because many phlorotannins present isomers. In addition, there are no available standards for all compounds, so data are presented as attempted identifications based on the literature. Semi-quantification was performed using a standard for each class of phenolic compounds and the detailed list of detected compounds can be found in Table 3. Molecules semi-quantified with concentrations lower than 5 µg/mL were marked as below the quantification limit (LOQ).

**Figure 2:**
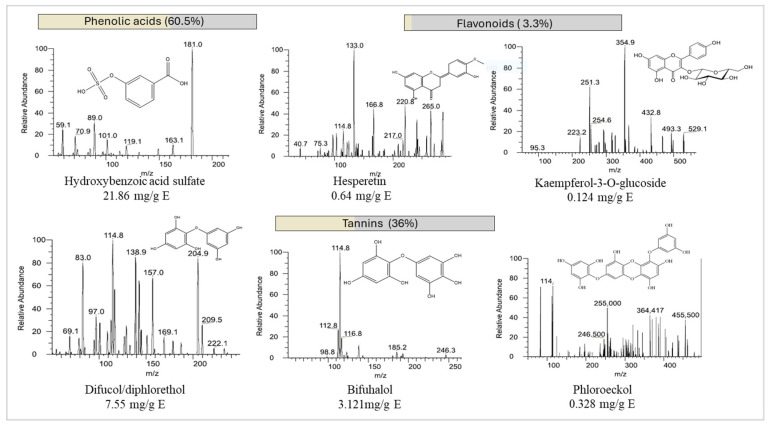
Mass spectra of the most abundant molecules in *SM* analyzed by HPLC-ESI-QqQ-MS/MS. Note: Please note for hydroxybenzoic acid sulfate, this is a representation of one of the possible isomeric forms.

As far as the phenolic acid family is concerned, its total contribution to the total phenolic content was estimated at 60.5%. The most significant molecule was hydroxybenzoic acid sulfate with a concentration of 21.86 mg/g E (Figure 2), isomeric forms were not considered. The presence of this molecule in SM was demonstrated previously in an ethyl acetate fraction as the most intense peak [73]. The second most abundant compound in the phenolic acid family was hydroxybenzoic acid glucoside, which was detected at the concentration of 0.258 mg/g E, with the precursor ion of [M − H]^−^ 316. This secondary metabolite is not typically detected in algae’s phenolic profiles but was previously detected by Zhong and colleagues in *Sargassum* sp. [51].

As for the flavonoid family, their contribution was estimated at 3.3%. Hesperetin was the molecule quantified in the highest quantity at 0.642 mg/g E ([M − H]^−^ 303 and MS^2^ 90.9 and 262.9). To our knowledge, there are no previous records of the presence of this molecule in SM. However, this flavonoid was found in the brown algae species *Bifurcaria bifurcata* and *Fucus spiralis* at concentrations of 70.35 and 66.4 µg/g E, corroborating our findings [54].

As expected, the group of tannins was the one with the highest number of tentatively identified compounds. Their contribution to the total detected phenolic compounds was estimated at 36%. A study made on SM (pressurized liquid extraction; EtOH:H_2_O) has tentatively identified several phlorotannins with different degrees of polymerization (from 3 to 11) by HPLC-MS; however, no quantification was made based on the chromatographic results [52]. In this study, a total of 43 tannins were tentatively identified. The most abundant phlorotannin was difucol [M − H]^+^ 251.097, quantified as 7.55 mg/g E, followed by bifuhalol [M − H]^+^ 266.592, with a concentration of 3.121 mg/g E. These two compounds constituted, based on phloroglucinol equivalents, approximately 80% of the total phlorotannins detected.

### 3.4. Biological Activities of the Optimum Extract

#### 3.4.1. Scavenging Activity of ROS and RNS

Free radicals are naturally formed as a metabolic consequence of cells’ reactions with oxygen. However, these processes are not the only source of oxidative stress, environmental pollution, UV radiation, exposure to pesticide residues, and cigarette smoke, among others, are responsible for an increase in these harmful molecules. Furthermore, oxidative stress that appears when ROS exceeds the cellular antioxidant system capacity can be related to pathologies like neurodegeneration, cardiovascular diseases, and cancer [74].

Consequently, antioxidants play a critical role in health and food preservation by constraining free radicals through scavenging mechanisms. To assess the macroalga extract’s scavenging capacity, several in vitro tests were performed against relevant ROS and RNS, such as the superoxide anion radical, hydrogen peroxide, and nitric oxide radical [75]. The SM extract’s antiradical scavenging capacity is presented in Table 4.

ROS occurs naturally in aerobic organisms as a metabolic product of oxygen. Likewise, the superoxide anion O_2_^•−^ can appear as a response of the immune system to pathogens or after the stimulation of the O_2_ molecule by irradiation. The non-radical species hydrogen peroxide is a part of several cellular mechanisms, and due to its relative stability and the high permeability of the cell membrane can induce toxicity, being commonly used as an oxidative stress promoter in in vitro models [8]. These species promote cellular damage and induce several human diseases [75]. These facts substantiate the importance of finding natural extracts with ROS scavenging capacity.

**Figure 3:**
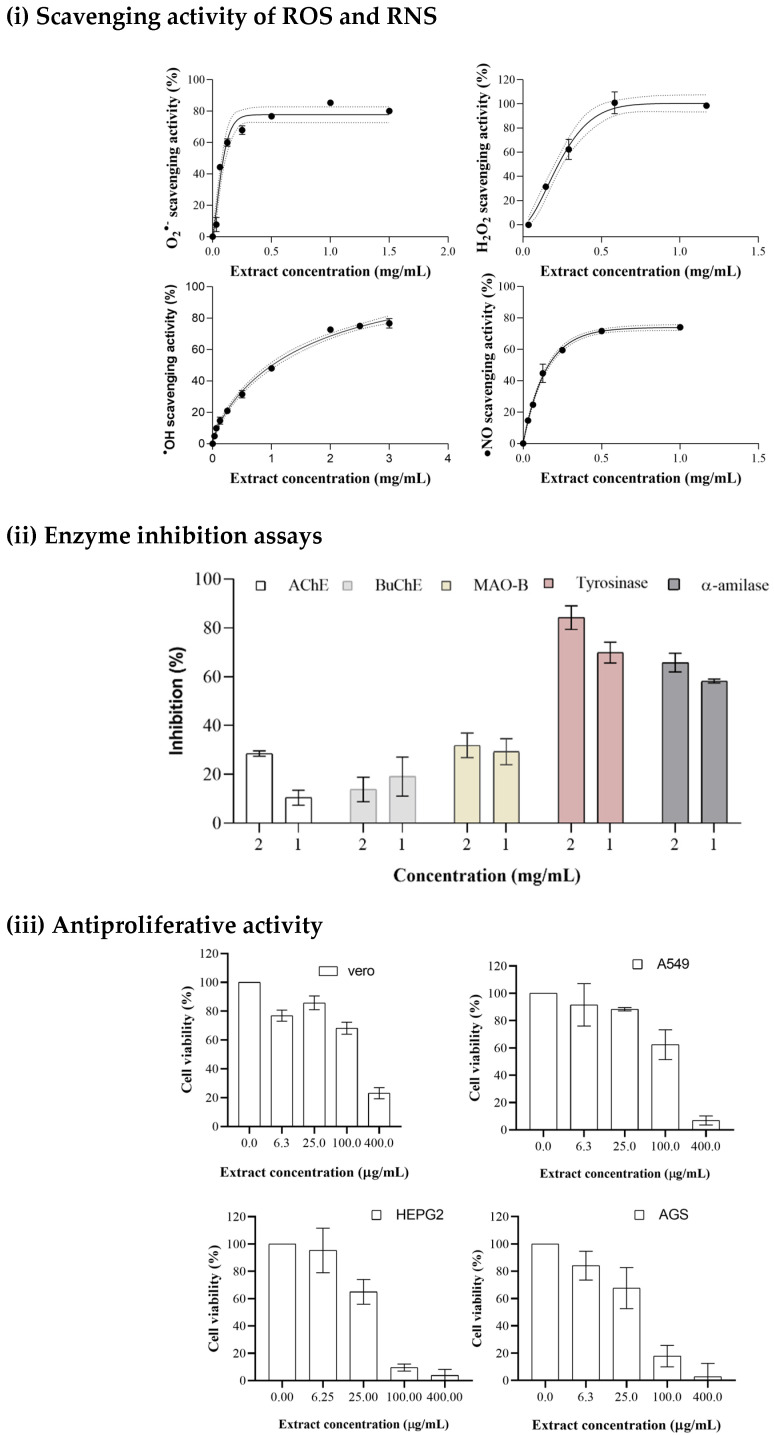
(**i**) Dose–response curves of superoxide anion radical, hydroxyl radical, hydrogen peroxide, and nitric oxide radical scavenging activities—dotted lines stand for 95% confidence levels. (**ii**) Inhibition response of enzyme tested at the extract concentrations of 2 and 1 mg/mL. (**iii**) Dose–response bars of A549, HepG2, AGS, and Vero cell inhibition rates—error bars represent the standard deviation; *n* = 3.

Figure 3 shows that the SM extract scavenges the oxidizing molecules in a dose-dependent manner. The performance of SM as a superoxide scavenger (IC_50_ = 57.72 µg/mL) is better than that of the reference molecule (IC_50_ = 160 µg/mL). The antioxidant capacity against the nitric oxide radical is more than four times greater than ascorbic acid, revealing the possibility of SM extract having anti-inflammatory properties, as there is a known link between the ●NO radical and inflammatory processes [76]. The high antioxidant potential of SM extract is related to the high concentration of phenolic acids [77] and phlorotannins [78], which are the major compounds of the SM phenolic profile, as supported by the cited literature. Moreover, these results may be justified by previous findings that Apo-9’-fucoxanthinone from SM effectively suppressed lipopolysaccharide-induced nitric oxide (^●^NO) [79]. It has also been related to cytoprotective characteristics by inhibiting hydrogen peroxide production and Caspase-9 enzyme activity [8].

#### 3.4.2. Antimicrobial Activity

SM extracts have been reported as a source of antimicrobial compounds [80]. Aiming to appraise the antimicrobial potential of the MAE-obtained extract, four foodborne pathogens, *B. cereus*, *E. coli*, *S. enteritidis*, and *P. aeruginosa*, and two microorganisms that cause opportunistic infections (*S. aureus* and *S. epidermidis*) were tested by the broth dilution method. The results revealed some antimicrobial activity against two of the tested species, *S. aureus* (8 mg/mL) and *P. aeruginosa* (8 mg/mL), and no substantial activity against the others.

The existing data on the antimicrobial activity of SM extracts are scarce. Acetone and chloroform extracts of SM have been reported to have some antimicrobial potential toward *Shigella fleschneri*, *Micrococcus* sp., and *Salmonella paratyphi* [81]. There is also a report of significant inhibition activity against *P. aeruginosa*, *E. coli*, and *S. aureus* achieved with SM acetone–water extracts [3]. Other authors described the nonexistence of antibacterial activity of SM extracts, suggesting that the presence of complex sugars such as polysaccharides in the sample triggered the growth of bacteria instead of repressing it [82].

#### 3.4.3. Inhibition of Enzymatic Activity

Neurodegenerative diseases are a major concern in this century, as populations are aging, and the prevalence of neurological disorders such as Alzheimer’s and Parkinson’s is also increasing [83]. Moreover, depression is a severe mental disorder that is a cause of disability worldwide and the most common comorbidity associated with neurodegenerative disorders [84]. Consequently, there is an upsurge in interest in natural products capable of interacting and potentially decreasing the occurrence of these diseases. The ability of the SM extracts to inhibit the AChE, BuChE, MAO-A, and MAO-B related to Alzheimer’s, Parkinson’s, and clinical depression disorders was researched. The results indicate a weak activity, with IC_50_ > 2 mg/mL of extract for all enzymes except for tyrosinase.

The data showed that 2 mg/mL of extract was able to promote a 28.5% inhibition rate of AChE; and a 19.5% inhibition effect on BuChE was observed in Figure 3. In another study, SM extracted with 1:1 methanol:dichloromethane solution led to no inhibition activity of SM extracts against AChE [85]. On the other hand, the result obtained for disruption of tyrosine activity was more significant, with an IC_50_ = 238.7 µg/mL (*p* < 0.05; *R*^2^ = 0.9756). This is a key enzyme associated with Parkinson’s disease, being involved in the increase in neuromelanin that leads to deficiency in the neurotransmitter dopamine and neuronal death [86,87].

Concerning MAO inhibition activity, an inhibition of 30% was achieved with the maximum extract concentration tested (2 mg/mL) towards MAO-B; however, no effect was found against MAO-A. In addition, the α-amylase inhibitory activity was also very significant, with an IC_50_ of 31.62 µg/mL (*p* < 0.05; *R*^2^ = 0.9114). There is already published work focusing on the inhibitory activity of carbohydrate-metabolizing enzymes by SM extracts, showing that a phlorotannin-rich extract (purified from a crude acetone–water (7:3, *v*/*v*) extract) of SM can inhibit the enzyme α-amylase to a moderate extent [88]. The result obtained in this work is much more effective for inhibiting α-amylase and highlights the effectiveness of the MAE extraction technique and the optimization procedure.

#### 3.4.4. Antiproliferative Activity

The ability of the SM extract, to inhibit the proliferation of abnormal cancer cells was tested on three cell lines, namely, A549 (adenocarcinoma of the lung), HepG2 (hepatocellular carcinoma), and AGS (adenocarcinoma of the stomach), as well as on kidney epithelial cells derived from African green monkey. The results show that SM extract effectively disrupts the proliferation of all tested cell lines depending on the dose (Figure 3), particularly against gastric and hepatocellular carcinoma (IC_50_ of 40.19 and 34.49 µg/mL, respectively). A thorough review demonstrated the capacity of individual or purified phlorotannin extracts to inhibit cancer cells [46]. Moreover, polyphenolic-rich methanolic extracts of SM have already shown efficiency in inducing cell death in human breast cancer cells [43]. In vivo tests in fertilized chicken eggs also reveal antiangiogenic activity [43]. Also, PLE-EtOH:H_2_O SM extract, as an antiproliferation agent of colorectal adenocarcinoma cells (HT-29 cells), has been determined to have a GI_50_ between 32.2 and 83.8 µg/mL, depending on the phlorotannin profile caused by the different samples’ origins, highlighting the importance of the phlorotannin content in the antiproliferation activity [52].

## 4. Conclusions

The RSM proved useful to optimize the MAE of *S. muticum* and resulted in the following optimal extraction conditions: time = 14.00 min; pressure = 11.03 bar; ethano*l* = 33.31%. Regarding the characterization of the phenolic compounds, the largest group was phenolic acids (60.5%) followed by phlorotannins (36%). The SM extract showed moderate activity in inhibiting enzymes associated with Alzheimer’s disease. The algal extract also inhibited MAO-B and demonstrated significant inhibitory activity against tyrosine and α-amylase enzymes (IC_50_ of 238.7 and 31.6 µg/mL, respectively). The obtained crude extract was evaluated for its antioxidant capacity and showed high activity against ROS and RNS, suggesting the possibility of using SM extract as a natural antioxidant additive. The crude extract studied exhibited cytotoxic properties against lung, liver, and gastric carcinoma cells. SM extract affected the growth of two of the six strains of microorganisms tested, *S. aureus* and *P. aeruginosa*, with estimated MICs of 8 mg/mL. These results allow us to suggest the application of SM extracts as antioxidant additives with nutraceutical characteristics.

## Figures and Tables

**Table 1:** Experimental RSM results of the CCCD for the MAE optimization of the independent variables (*X*_1_, *X*_2_, and *X*_3_) for the five assessed responses (*yield*, *TPC*, *DPPH*, *ABTS*, and *BCM*). Variables are presented in natural values and codified ranges.

	Experimental Design						Response				
	Coded Value			Natural Value							
	*X_1_*	*X_2_*	*X_3_*	*X_1_*: *t*	*X_2_*: *P*	*X_3_*: *S*	*Y*	TPC	DPPH	ABTS	BCM
				min	Bar	%	mg/g dw	mg PGE/g dw	nM R•/g dw	nM R•/g dw	µM βC/g dw
1	−1	−1	−1	7.5	5.6	20.3	408.113	52.949	32.613	30.013	0.077
2	−1	−1	1	7.5	5.6	79.7	284.507	35.267	22.206	43.618	0.019
3	−1	1	−1	7.5	16.4	20.3	516.582	73.520	40.914	34.242	0.076
4	−1	1	1	7.5	16.4	79.7	329.433	39.579	24.805	50.334	0.025
5	1	−1	−1	20.5	5.6	20.3	447.439	54.063	29.799	26.433	0.103
6	1	−1	1	20.5	5.6	79.7	319.175	39.136	24.985	48.838	0.037
7	1	1	−1	20.5	16.4	20.3	477.789	72.839	36.781	31.324	0.050
8	1	1	1	20.5	16.4	79.7	327.477	40.152	14.969	43.802	0.015
9	1.68	0	0	25	11	50	422.345	43.478	23.240	90.862	0.028
10	−1.68	0	0	3	11	50	339.210	47.617	23.277	88.618	0.062
11	0	−1.68	0	14	2	50	283.555	31.099	16.772	37.657	0.039
12	0	1.68	0	14	20	50	416.642	39.453	20.731	46.387	0.035
13	0	0	−1.68	14	11	0	412.876	27.954	13.193	21.225	0.095
14	0	0	1.68	14	11	100	90.659	14.862	13.383	26.439	0.108
15	−1.68	−1.68	−1.68	3	2	0	326.108	14.007	6.283	14.382	0.049
16	−1.68	−1.68	1.68	3	2	100	45.433	9.453	4.951	11.464	0.002
17	−1.68	1.68	−1.68	3	20	0	408.116	26.199	11.739	40.126	0.035
18	−1.68	1.68	1.68	3	20	100	92.315	14.401	11.175	19.584	0.007
19	1.68	−1.68	−1.68	25	2	0	411.209	15.708	3.755	21.712	0.033
20	1.68	−1.68	1.68	25	2	100	64.104	2.232	3.506	6.091	0.010
21	1.68	1.68	−1.68	25	20	0	434.463	27.583	14.425	25.247	0.036
22	1.68	1.68	1.68	25	20	100	111.355	6.944	10.739	22.927	0.004
23	0	0	0	14	11	50	342.489	45.218	51.749	51.096	0.063
24	0	0	0	14	11	50	359.681	42.236	62.114	52.667	0.077
25	0	0	0	14	11	50	381.981	33.916	53.071	39.287	0.051
26	0	0	0	14	11	50	407.065	41.149	58.712	66.833	0.084
27	0	0	0	14	11	50	393.724	38.906	61.215	49.374	0.070
28	0	0	0	14	11	50	364.892	37.272	65.683	65.124	0.062

**Table 2:** Parametric results of the polynomial fitting of Equation (1) for MAE and in terms of the extraction behavior for the five assessed responses (yield, TPC, DPPH, ABTS, and BCM) (A). Variables are presented in codified ranges and the parametric subscripts 1, 2, and 3 refer to the variables involved (*X*_1_, *X*_2_, and *X*_3_, respectively). Statistical information of the fitting analysis is also shown. (B) Optimum conditions in natural values that lead to optimal response values.

Coefficients		Parametric Responses to the cccd				
		Extract	Phytochemical	Antioxidant Activity		
		Yield	TPC	DPPH^•^	ABTS^•+^	BCM
**(A) Fitting Coefficients Obtained**						
*Intercept*	*b*_0_	379.21 ± 17.58	41.62 ± 3.90	58.92 ± 3.62	53.86 ± 5.0	0.070 ± 0.005
*Linear* * effect*	*b*_1_	*ns*	*ns*	*ns*	*ns*	*ns*
	*b*_2_	20.70 ± 8.51	3.18 ± 1.89	1.77 ± 1.47	*ns*	−0.013 ± 0.010
	*b*_3_	−62.28 ± 28.54	−17.12 ± 6.34	*ns*	13.66 ± 6.8	−0.037 ± 0.010
*Quadratic* *effect*	*b*_11_	14.13 ± 13.70	5.24 ± 3.04	−10.72 ± 2.39	10.26 ± 3.3	−0.011 ± 0.004
	*b*_22_	*ns*	*ns*	−12.31 ± 2.39	−6.61 ± 3.3	−0.014 ± 0.004
	*b*_33_	−31.48 ± 13.70	−3.30 ± 3.04	−14.25 ± 2.39	−13.05 ± 3.3	0.009 ± 0.004
*Cubiceffect*	*b*_111_	*ns*	*ns*	*ns*	*ns*	*ns*
	*b*_222_	*ns*	*ns*	*ns*	*ns*	0.004 ± 0.004
	*b*_333_	−11.38 ± 11.23	4.72 ± 2.49	*ns*	−5.59 ± 2.7	0.011 ± 0.004
*Interactive* *effect*	*b*_12_	*ns*	*ns*	*ns*	*ns*	*ns*
	*b*_13_	*ns*	*ns*	*ns*	*ns*	*ns*
	*b*_23_	*ns*	*ns*	*ns*	*ns*	*ns*
	*b*_123_	*ns*	*ns*	*ns*	*ns*	*ns*
	*b*_1122_	*ns*	*ns*	*ns*	*ns*	*ns*
	*b*_1133_	*ns*	*ns*	*ns*	*ns*	*ns*
	*b*_2233_	*ns*	*ns*	*ns*	*ns*	*ns*
	*b*_112233_	−4.07 ± 2.22	−1.42 ± 0.49	2.43 ± 0.49	*ns*	*ns*
	*R* ^2^	0.9298	0.8140	0.9042	0.8289	0.8487
**(B) Optimal Conditions And Response Values Obtained**						
Optimum conditions	*X*_1_: *t* (*min*)	14.00 ± 1.87	3.00 ± 0.87	14.00 ± 1.87	3.00 ± 0.87	14.00 ± 1.87
	*X*_2_: *P* (*bar*)	20.00 ± 2.24	20.00 ± 2.24	11.38 ± 1.69	11.00 ± 1.66	8.90 ± 1.49
	*X*_3_: *S* (*%*)	0.00 ± 0.00	35.88 ± 3.0	50.00 ± 3.54	61.72 ± 3.93	8.70 ± 1.47
		*mg*/*g dw*	*mg*/*g dw*	*nM R^•^*/*g dw*	*nM R^•^*/*g dw*	*µM βC*/*g dw*
Response		483.87 ± 35.26	66.10 ± 13.03	58.98 ± 17.30	85.88 ± 20.70	0.112 ± 0.043
Experimental		469.4 ± 8.86	44.28 ± 1.95	50.15 ± 2.84	83.20 ± 11.57	0.101 ± 0.096

Abbreviations: *ns*: non-significant coefficient; *R*^2^: coefficient of determination.

**Table 3:** Annotated phenolic compounds in *SM* analyzed by HPLC-ESI-QqQ-MS/MS.

ID	Ref	Pol	Formula	MW (Da)	Prec. (*m/z*)	Product (*m/z*)	Col. Energy (V)	RFL (V)	Compound	Class	Subclass	mg/g E
C1	[51]	[M − H]^−^	C_16_H_16_O_6_	304.095	302.72	92.883, 94.8	17.54, 17.43	111	3-*O*-Methylcatechin	Flavonoids	Flavanols	0.029
C2	[51]	[M − H]^−^	C_15_H_14_O_7_	306.074	322.877	138.967, 240.883	27.39, 18.41	109	Gallocatechin	Flavonoids	Flavanols	<LOQ
C3	[53,54]	[M − H]^+^	C_16_H_14_O_6_	302.079	303.636	90.967, 262.967	27.44, 5.3	117	Hesperetin	Flavonoids	Flavanones	0.642
C4	[55,56]	[M − H]^−^	C_21_H_22_O_11_	450.116	448.576	94.8, 300.717	28.35, 9.14	104	Taxifolin-*O*-rhamnoside	Flavonoids	Flavanonols	0.010
C5	[49]	[M − H]^+^	C_16_H_12_O_5_	284.068	306.358	90.967, 91.967	28.3, 28.71	97	Acacetin	Flavonoids	Flavones	<LOQ
C6	[47,57]	[M − H]^−^	C_21_H_20_O_10_	432.106	448.659	266.8, 300.717	10.11, 9.2	86	Apigenin-7-glucoside	Flavonoids	Flavones	0.021
C7	[49]	[M − H]^−^	C_16_H_12_O_6_	300.063	298.689	92.917, 224.667	18, 9	105	Hispidulin	Flavonoids	Flavones	0.070
C8	[47]	[M − H]^+^	C_21_H_20_O_11_	448.101	470.64	112.8, 336.717	26.58, 10.56	108	Luteolin-*O*-hexoside	Flavonoids	Flavones	0.125
C9	[58]	[M − H]^+^	C_27_H_30_O_16_	610.153	610.544	112.8, 246.717	36.69, 25.62	147	Luteolin-7-*O*-rutinoside	Flavonoids	Flavones	0.063
C10	[51]	[M − H]^−^	C_27_H_30_O_14_	578.164	576.706	352.55, 394.717	18.85, 8.39	93	Rhoifolin	Flavonoids	Flavones	<LOQ
C11	[51]	[M − H]^−^	C_17_H_14_O_7_	330.074	346.794	138.667, 272.833	15, 9	68	3,7-Dimethylquercetin	Flavonoids	Flavonols	0.007
C12	[59,60]	[M − H]^+^	C_22_H_22_O_12_	478.111	478.668	112.8, 114.8	24.46, 23.45	143	Isorhamnetin 3-*O*-glucoside	Flavonoids	Flavonols	0.090
C13	[47,61]	[M − H]^+^	C_21_H_20_O_11_	448.101	470.64	112.8, 114.8	26.63, 26.84	109	Kaempferol-3-*O*-glucoside	Flavonoids	Flavonols	0.125
C14	[47]	[M − H]^−^	C_27_H_30_O_16_	610.153	626.457	285, 552.583	25, 13	102	Kaempferol-*O*-hesoxide	Flavonoids	Flavonols	<LOQ
C15	[47,57]	[M − H]^−^	C_27_H_30_O_15_	594.158	628.428	446.633, 554.633	11.07, 12.68	98	Kaempferol-*O*-rutinoside	Flavonoids	Flavonols	0.009
C16	[62]	[M − H]^−^	C_15_H_10_O_8_	318.038	316.997	151, 179	24, 19	161	Myrecetin	Flavonoids	Flavonols	0.007
C17	[47]	[M − H]^−^	C_21_H_19_O_12_	463.088	462.63	271, 300.167	43, 27	119	Quercetin-*O*-glucoside	Flavonoids	Flavonols	<LOQ
C18	[47,61]	[M − H]^−^	C_24_H_22_O_15_	550.096	566.899	251.25, 354.917	37, 15	119	Quercetin-3-*O*-malonylglucoside	Flavonoids	Flavonols	<LOQ
C19	[47]	[M − H]^−^	C_27_H_30_O_16_	610.153	609.113	271.083, 300.33	59, 37	291	Quercetin-*O*-rutinoside	Flavonoids	Flavonols	<LOQ
C20	[51]	[M − H]^−^	C_22_H_20_O_11_	460.101	494.37	270.8, 344.8	19.3, 11.12	113	Glycitein-7-*O*-glucuronide	Flavonoids	Isoflavone	<LOQ
C21	[51]	[M − H]^−^	C_17_H_16_O_5_	300.1	316.736	92.883, 94.8	16.32, 16.27	88	Sativanone	Flavonoids	Isoflavone	<LOQ
C22	[51]	[M − H]^−^	C_7_H_6_O_2_	122.037	156.889	70.967, 96.883	16.63, 12.99	79	*p*-Hydroxybenzaldehyde	Other	HBD	0.018
C23	[51]	[M − H]^−^	C_10_H_8_O_4_	192.042	190.918	86.917, 110.917	17, 12	48	Scopoletin	Other	HC	0.077
C24	[51]	[M − H]^−^	C_20_H_26_O_4_	330.183	328.893	271, 293, 271, 293	14, 12, 14, 12	72	Carnosol	Other	Other	<LOQ
C25	[51]	[M − H]^−^	C_13_H_16_O_8_	300.085	316.721	92.8, 94.8	16.27, 16.52	90	4-Hydroxybenzoic acid glucoside	Phenolic acids	HBA	0.258
C26	[62]	[M − H]^−^	C_7_H_6_O_5_	170.022	168.981	79.24, 125.083	24, 15	121	Gallic acid	Phenolic acids	HBA	0.038
C27	[63]	[M − H]^−^	C_7_H_6_O_6_S	217.989	217.031	88.967, 181.05	20.11, 9.4	62	Hydroxybenzoic acid sulphate	Phenolic acids	HBA	21.861
C28	[51]	[M − H]^−^	C_8_H_8_O_7_S	247.999	247.042	88.967, 211.05	22, 10.81	150	Vanillic acid 4-sulfate	Phenolic acids	HBA	0.047
C29	[51]	[M − H]^−^	C_16_H_18_O_9_	354.095	353.055	111, 172.917	22, 12	83	Chlorogenic acid	Phenolic acids	HCA	<LOQ
C30	[64]	[M − H]^−^	C_7_H_12_O_6_	192.063	191.023	86.917, 110.917	17, 12	76	Quinic acid	Phenolic acids	HCA	0.110
C31	[51]	[M − H]^−^	C_11_H_12_O_5_	224.068	222.944	164.883, 204.717	13.09, 13.49	150	Sinapic acid	Phenolic acids	HCA	<LOQ
C32	[65]	[M − H]^+^	C_12_H_10_O_7_	266.043	266.592	112.833, 114.833	9, 8	84	Bifuhalol	Tannins	HT	3.121
C33	[66]	[M − H]^−^	C_24_H_18_O_12_	498.08	496.706	92.833, 240.833	32, 18	75	Bisfucophlorethol	Tannins	HT	0.047
C34	[65]	[M − H]^−^	C_36_H_22_O_18_	742.081	758.834	626.583, 684.75	14, 15	113	Dieckol	Tannins	HT	0.013
C35	[50,65]	[M − H]^+^	C_12_H_10_O_6_	250.048	251.097	82.833, 205.083	33, 16	65	Difucol	Tannins	HT	7.555
C36	[47]	[M − H]^−^	C_20_H_20_O_14_	484.085	482.545	332.717, 334.717	10.11, 10.71	119	Digalloylglucose	Tannins	HT	0.039
C37	[49]	[M − H]^−^	C_24_H_18_O_13_	514.075	548.594	338.8, 474.633	19.86, 11.87	90	Deshydroxetrafuhalol	Tannins	HT	<LOQ
C38	[49]	[M − H]^−^	C_42_H_30_O_23_	902.118	900.411	616.633, 632.383	21.68, 30.48	147	Dihydroxiheptafuhalol	Tannins	HT	<LOQ
C39	[52]	[M − H]^+^	C_36_H_26_O_19_	762.107	784.646	183.967, 602.633	29.97, 8.44	154	Dihydroxyhexafuhalol	Tannins	HT	0.075
C40	[52]	[M − H]^+^	C_54_H_38_O_28_	1134.155	1156.444	974.383, 1065.383	10.66, 652	179	Dihydroxynonafuhalol	Tannins	HT	0.088
C41	[52]	[M − H]^−^	C_48_H_34_O_25_	1010.139	1008.432	528.05, 972.55	39.27, 15.11	299	Dihydroxyoctafuhalol	Tannins	HT	0.013
C42	[52]	[M − H]^+^	C_30_H_22_O_16_	638.091	638.398	112.8, 456.717	38.72, 10.31	133	Dihydroxypentafuhalol	Tannins	HT	0.041
C43	[52]	[M − H]^−^	C_60_H_42_O_31_	1258.171	1275.057	1184.3	5.61	165	Deshydroydecafuhalol	Tannins	HT	0.034
C44	[65]	[M − H]^+^	C_18_H_10_O_9_	370.032	370.582	112.8, 114.717	16.78, 18.65	94	Dioxinodehydroeckol	Tannins	HT	0.360
C45	[67]	[M − H]^−^	C_24_H_16_O_13_	512.059	510.602	210.8, 284.8	24.87, 17.89	97	Diphlorethohydroxycarmalol	Tannins	HT	0.037
C46	[65]	[M − H]^+^	C_18_H_12_O_9_	372.048	371.348	112.917, 118.833	15, 5	104	Eckol	Tannins	HT	0.070
C47	[66]	[M − H]^−^	C_24_H_18_O_12_	498.08	496.623	287.083, 348.667	18, 9	103	Fucodiphlorethol	Tannins	HT	0.024
C48	[68]	[M − H]^−^	C_18_H_10_O_9_	370.032	368.825	110.883, 186.8	20.72, 10.56	70	Phloroethol	Tannins	HT	0.328
C49	[66]	[M − H]^−^	C_18_H_14_O_9_	374.064	372.774	224.75, 298.17	14, 9	93	Fucophlorethol	Tannins	HT	<LOQ
C50	[69]	[M − H]^−^	C_60_H_42_O_30_	1242.176	1241.302	877.383, 968.383	24, 16	174	Fucophlorethol decamer	Tannins	HT	<LOQ
C51	[70]	[M − H]^−^	C_42_H_30_O_21_	870.128	869.421	589, 792.833, 794.5	25, 17, 14	127	Fucophlorethol heptamer	Tannins	HT	<LOQ
C52	[70]	[M − H]^−^	C_36_H_26_O_18_	746.112	744.405	596.25, 670.5	19, 12	119	Fucophlorethol hexamer	Tannins	HT	<LOQ
C53	[69]	[M − H]^−^	C_54_H_38_O_27_	1118.16	1153.262	971.467, 1135.55	12.08, 9.35	149	Fucophlorethol nonamer	Tannins	HT	0.038
C54	[69]	[M − H]^−^	C_48_H_34_O_24_	994.144	992.437	810.467, 901.467	10.51, 7.68	142	Fucophlorethol octamer	Tannins	HT	0.059
C55	[66]	[M − H]^−^	C_36_H_26_O_18_	746.112	744.405	460.55, 670.55	23.55, 12.99	113	Fucotetraphlorethol	Tannins	HT	0.026
C56	[70]	[M − H]^−^	C_30_H_22_O_15_	622.096	656.365	506.633, 582.55	14.25, 12.63	99	Fucotriphlorethol	Tannins	HT	0.087
C57	[71]	[M − H]^−^	C_42_H_30_O_21_	870.128	904.397	550.55, 624.633	31.99, 22.19	127	Heptafucol	Tannins	HT	<LOQ
C58	[65]	[M − H]^−^	C_42_H_30_O_24_	918.113	952.382	672.133, 878.383	51, 17.84	256	Heptafuhalol	Tannins	HT	0.020
C59	[52]	[M − H]^+^	C_36_H_26_O_21_	794.097	832.443	773.55, 814.467	26.08, 5.3	148	Hexafuhalol	Tannins	HT	<LOQ
C60	[47]	[M − H]^+^	C_42_H_30_O_25_	934.108	934.415	722.883, 752.467	23.55, 12.13	151	HHDP-galloylglucose	Tannins	HT	0.177
C61	[66]	[M − H]^−^	C_24_H_14_O_12_	494.049	492.591	344.717, 418.717	11.22, 10.71	113	Hydroxyfucofuroeckol	Tannins	HT	0.045
C62	[52]	[M − H]^+^	C_42_H_30_O_25_	934.108	934.415	752.467, 850.467	11.42, 16.78	152	Hydroxyheptafuhalol	Tannins	HT	0.182
C63	[49]	[M − H]^−^	C_36_H_26_O_22_	810.092	808.384	658.55, 676.55	13.34	116	Hydroxyhexafuhalol	Tannins	HT	<LOQ
C64	[49]	[M − H]^−^	C_30_H_22_O_18_	670.081	668.54	486.6, 520.6, 594.6	10.16, 14.1, 13.8	103	Hydroxypentafuhalol	Tannins	HT	0.059
C65	[71]	[M − H]^−^	C_24_H_18_O_15_	546.065	580.501	300.8, 506.633	13.14, 12.03	99	Hydroxytetrafuhalol	Tannins	HT	0.061
C66	[52]	[M − H]^−^	C_48_H_34_O_28_	1058.124	1056.749	844.717	14.65	153	Octafuhalol	Tannins	HT	<LOQ
C67	[71]	[M − H]^−^	C_30_H_22_O_17_	654.086	652.545	262.717, 470.633	29.72, 9.95	101	Pentafuhalol	Tannins	HT	<LOQ
C68	[47]	[M − H]^+^	C_24_H_16_O_12_	496.064	496.614	112.917, 114.833	23, 22	133	Phloroeckol	Tannins	HT	0.244
C69	[51]	[M − H]^+^	C_6_H_6_O_3_	126.032	126.081	56.083, 98	21, 12	78	Phloroglucinol	Tannins	HT	0.053
C70	[72]	[M − H]^−^	C_24_H_18_O_14_	530.07	528.612	394.667, 454.667	10, 11	110	Tetrafuhalol	Tannins	HT	0.009
C71	[69]	[M − H]^−^	C_18_H_14_O_9_	374.064	372.69	93224.833	23, 15	93	Trifucol	Tannins	HT	0.139
C72	[52]	[M − H]^−^	C_18_H_14_O_10_	390.059	388.768	206.8333, 14.75	9, 10	68	Trifuhalol	Tannins	HT	0.040
C73	[65]	[M − H]^−^	C_30_H_20_O_17_	652.07	650.613	318.717, 468.633	19.1, 9.75	101	Trifuhalolhydroxycarmalol	Tannins	HT	<LOQ
C74	[51]	[M − H]^−^	C_13_H_8_O_4_	228.042	244.929	94.8, 96.717	6.47, 6.01	63	Urolithin A	Tannins	HT	0.071

Abbreviations: MW: molecular weight, Prec.: precursor ion, HBAs: hydroxybenzoic acids; HCAs: hydroxycinnamic acids; HTs: hydrolzable tannins; HBDs: hydroxybenzaldehydes; HCs: hydroxycoumarins. The chemical compounds detected were classified into the following classes: alkaloids, flavonoids, phenolic acids, flavonoids, tannins, and terpenes. The semi-quantification of these classes was determined according to the calibration curves of the following standards, respectively: caffeine (*y =* 610,791 *x*), epigallocatechin (*y =* 65,138 *x*), gallic acid (*y =* 12,778 *x*), ferulic acid (*y =* 138,538 *x*), phloroglucinol (*y =* 13,888 *x*), and genipin (*y =* 76,564 *x*). Molecules semi-quantified in concentrations lower than 5 µg/mL were marked as below the quantification limit (LOQ). Pol: polarity; MW: molecular weight; Prec.: precursor; Col. Energy: collision energy; RFL: RF lens.

**Table 4:** Bioactivity analyses of *SM* optimized extract. (A) Antioxidant activity; (B) inhibition of central nervous system-related enzymes; (C) antiproliferative effects; (D) antimicrobial activity against Gram-negative and Gram-positive pathogenic bacteria.

	Optimized Extract	Positive Control
**A: Antioxidant Activity (IC_50_, µg/mL)**		
	IC_50_ (µg/mL)	Ascorbic acid
^•^NO	100.1	446
O_2_^−•^	57.72	160
H_2_O_2_	227.9	51
OH^•−^	989.5	183
**B: Health promoting enzymes (IC_50_, µg/mL)**		
tyrosinase	238.7	Kojic acid = 2.00
α-amylase	31.60	Acarbose = 300
**C: Cytotoxicity (GI_50_, µg/mL)**		
		Ellipticine
A549 (lung adenocarcinoma)	132.7	<0.78
HepG2 (hepatocellular carcinoma)	34.49	0.85 ± 0.046
AGS (gastric adenocarcinoma)	40.19	<0.78
Vero	144.8	<0.78
**D: Antimicrobial activity (mm; mg/mL)**		
	Inhibition zone (mm)	MIC (mg/mL)
*Escherichia coli*	-	>8
*Staphylococcus epidermidis*	-	>8
*Bacillus cereus*	-	>8
*Staphylococcus aureus*	9.42 ± 1.04	8
*Salmonella enteritidis*	-	8
*Pseudomonas aeruginosa*		>8

Abbreviations: MIC, minimum inhibitory concentration; Vero, the African green monkey kidney-derived cell line; AGS, the human gastric cancer cell line; A549, the human lung adenocarcinoma cell line; HepG2, the human hepatocarcinoma cell line. IC_50_—half maximal effective concentration; the IC_50_ values were determined by fitting the experimental data (n = 3) to the Weibull model with a confidence level of 95% and an R^2^ > 0.9.

## Data Availability

The original data presented in the study are included in the article; further inquiries can be directed to the corresponding author.
